# Progress of Epidemics in Twenty-Eight Towns and Districts

**Published:** 1856-04

**Authors:** 


### PROGRESS OF EPIDEMICS.

### LOCAL REPORTS OF EPIDEMIC AND ENDEMIC DISEASES

*During the Months of Dec., Jan., and Feb.* 1855-6.

| Place. | County. | Lat. | Long. | Observer. |
|---|---|---|---|---|
| Teignmouth - | Devonshire - | 50.32 N. | 3.29 W. | W. C. Lake, Esq. |
| Odiham - - | Hampshire - | 51. 8 N. | 1. 3 W. | J. M'Intyre, M.D. |
| Bridgewater - | Somerset - | 51. 8 N. | 3. 0 W. | Alfred Haviland, Esq. |
| Canterbury - | Kent - - | 51.17 N. | 1. 4 E. | G. Rigden, Esq.<br>James Reid, Esq. |
| Chatham - | Kent - - | 51.24 N. | 0.14 E. | F. J. Brown, M.D. |
| Putney - - | Surrey - - | 51.28 N. | 0. 3 W. | R. H. Whiteman, Esq. |
| Up. Holloway | Middlesex - | 51.32 N. | 0.03 E. | W. B. Kesteven, Esq. |
| Wanstead - | Essex - - | 51.32 N. | 0. 2 W. | F. Collins, M.D. |
| Swansea - | Glamorgansh. | 51.38 N. | 3.50 W. | W. H. Michael, Esq. |
| Saffron Walden | Essex - - | 52. 3 N. | 0.12 E. | T. Spurgin, Esq.<br>H. Stear, Esq. |
| Bedford - - | Bedfordshire - | 52. 8 N. | 1.51 W. | T. H. Barker, M.D. |
| Sharnbrook - | Bedfordshire - | | | R. S. Stedman, Esq. |
| Wellingbro' - | Northamptons. | 52.10 N. | 0.40 W. | B. Dulley, Esq. |
| Thetford - | Norfolk - - | 52.26 N. | 0.45 E. | H. W. Bailey, Esq. |
| East Dereham | Norfolk - - | 52.40 N. | 0.57 E. | J. Vincent, M.D. |
| Pontesbury - | Shropshire - | 52.43 N. | 2.50 W. | Wm. Eddowes, Esq. |
| Nottingham - | Nottinghamsh. | 52.50 N. | 1.10 W. | T. Robertson, M.D. |
| Burton-on-Trt. | Staffordshire - | 52.53 N. | 1.53 W. | S. Thomson, M.D. |
| Wrexham - | Denbighshire - | 53. 2 N. | 3. 1 W. | E. Williams, M.D. |
| Hawarden - | Flintshire - | 53.11 N. | 3. 2 W. | T. Moffat, M.D. |
| Lincoln - | Lincolnshire - | 53.12 N. | 0. 5 W. | S. Lowe, Esq. |
| Alford - - | Lincolnshire - | 53.15 N. | 0. 6 E. | R. U. West, M.D. |
| Gainsborough | Lincolnshire - | 53.23 N. | 0.47 W. | D. Mackinder, M.D. |
| Liverpool - | Lancashire - | 53.24 N. | 2.59 W. | Thos. Bickerton, Esq. |
| Bolton - - | Lancashire - | 53.35 N. | 2.19 W. | W. H. Pendlebury, Esq. |
| Wst. Auckland | Durham - | 54.45 N. | 1.40 W. | G. Todd, Esq. |
| Rothbury - | Northumberld. | 55.25 N. | 1.50 W. | E. C. Summers, Esq. |

### QUARTERLY STATEMENT—No. V.

[*The dates denote that the disease appeared in the weeks then ending.*]

SCARLET FEVER.

Teignmouth..Dec. 14, Jan. 4. [8-15
Canterbury..Dec. 28, Jan. 4-25, Feb.
Chatham..All Jan. & nearly all Feb.
Putney..All December.
Upper Holloway..Feb. 8
Swansea..Dec. 7-21, Jan. 18, Feb. 1, 15
Saffron Walden..All Dec. and Jan.
Bedford..Dec. 7, Jan. 11-18
Sharnbrook..Dec. 21-28, Jan. 4, 18, Feb. 15-29
Wellingbro'..Every week to Feb. 15
Thetford..All Dec., Jan. 4-11, Feb. 15-29
East Dereham..Nearly all Jan. & Feb.
Pontesbury..Dec. 14-21 [8, 29
Nottingham..Every week except Feb.
Burton-on-Trent..Feb. 1, 15-22
Wrexham..Every week
Lincoln..Feb. 15 29
Alford..Jan. 25, Feb. 22
Liverpool..Every week
Bolton..Every week

MEASLES.

Bridgewater..Jan. 4, 11
Canterbury..Every week
Chatham..Dec. 14, Jan. 11-25, Feb. [15-29
Swansea..Dec. 14, Jan. 4
Wellingborough..All Feb.
Thetford..Every week

Nottingham..All Feb.
Wrexham..Dec. 28, Jan. 4-18
Hawarden..Dec. 28, Jan. 4
Liverpool..Every week
Bolton..Dec.21-28,Jan.4,25,Feb.1-15
Rothbury..Jan. 4

SMALL-POX.

Canterbury..Every week
Chatham..Jan, 18-25, Feb. 1, 15, 29
Putney..Feb. 15-22
Upper Holloway..Jan. 11
Bedford..Dec. 7, 21, Feb. 8-29
Wellingborough..Feb. 22-29
Nottingham..Dec. 7
Alford..Feb. 15-22
Liverpool..Every week

HOOPING COUGH.

Odiham..Dec. 28, all Jan. and Feb.
Canterbury..Every week
Chatham..Feb. 1, 22, 29
Putney..All Jan. and Feb.
Upper Holloway..Jan. 18-25
Wanstead..Jan. 25, Feb. 1, 15
Saffron Walden..Every week
Sharnbrook..Feb. 29
Thetford..All Dec. and Jan.
East Dereham..All Dec. and Jan.
Nottingham..Every week
Burton-on-Trent..Jan. 25, Feb. 29
Hawarden..Jan. 18-25
Lincoln..All Dec.
Alford..Feb. 15-29
Gainsborough..Every week
Liverpool..Every week
Bolton..Dec.21-28,Jan.4,25,Feb.1-22

CROUP.

Teignmouth..Dec. 7
Odiham..Feb. 1, 15
Bridgewater..Jan. 18
Canterbury..Dec. 21-28, Jan. 18
Wanstead..Dec. 28
Wellingborough..Feb. 15
Thetford..Dec. 21
Nottingham..Dec. 14, Jan. 5
Burton-on-Trent..Feb. 8
Alford..Feb. 1
Gainsborough..Dec.7,Jan.11-25,Feb.1
Liverpool..Feb. 29
Bolton..Jan. 11-18

CATARRH.

Teignmouth..All Dec. & Jan., Feb. 1,
Odiham..Every week [8, 29
Bridgewater..Dec. 28, Jan. 4
Canterbury..Every week
Chatham..Every week
Putney..Every week except Dec. 14
Up. Holloway..Jan. 4, 25, Feb. 1, 15, 22
Wanstead..All Dec. and Jan.
Swansea..Every week
Saffron Walden..Every week
Bedford..Dec. 7, 28
Sharnbrook..Dec. 21-8, all Jan., Feb. 1
Wellingborough..Every week
Thetford..Dec. 7, 21, 28, Jan. 11-25, Feb. 1, 8, 22, 29
East Dereham..All Feb.
Pontesbury..Dec. 14-28, Feb. 1-22
Nottingham..Every week
Burton-on-Trent..All Feb.
Hawarden..Feb. 8
Gainsborough..Every week
Liverpool..Every week
Bolton..Dec.14-28,Jan.11-25,Feb.1,8
Rothbury..Every week

INFLUENZA.

Teignmouth..All Dec. & Jan., Feb. 1, 15, 29
Odiham..Every week except Feb. 22, 29
Bridgewater..Jan. 4
Chatham..Dec. 21, 28, Jan. 4, 11, Feb.
Putney..Jan. 25, all Feb. [1-22
Wanstead..All Dec. & Jan., Feb. 1, 8.
Swansea..Feb. 1-29
Saffron Walden..Dec.28, all Jan.&Feb.
Bedford..Every week except Jan. 25 and Feb. 29
Sharnbrook..Dec. 28, Jan. 18
Wellingborough..All Jan. & Feb.
Thetford..All Dec., Jan. 4, 18, 25, Feb. 1, 15, 29
Pontesbury..Feb. 15, 22
Nottingham..All Dec. & Jan., Feb. 29
Burton-on-Trent..Feb. 8-22
Lincoln..Feb. 1-22
Alford..Dec. 21
Gainsborough..Dec. 28, all Jan.
Liverpool..Dec. 14, Jan. 4, 18, 25, Feb.
Bolton..Jan. 18, 25, Feb. 1 [8, 29

ERYSIPELAS.

Teignmouth..Feb. 8
Odiham..Dec. 21, Feb. 1, 15
Canterbury..Jan. 4, 25, Feb. 1, 8, 22
Chatham..Dec.14,21,Jan.4-18,Feb.15
Putney..Feb. 8
Wanstead..Dec. 7-21, Jan. 4, Feb. 8.
Swansea..Jan. 11, Feb. 15-29
Saffron Walden..Jan. 11, 25, Feb. 15
Bedford..Dec. 7, Feb. 1-15
Sharnbrook..Dec. 7, Jan. 4, 18
Wellingborough..Jan. 25, Feb. 1-15
Thetford..Dec. 14-28, Jan. 11, 25, Feb. 15-29
Pontesbury..Jan. 25, Feb. 1-22
Nottingham..Dec. 14-28, Jan. 11-25, Feb. 1-29
Wrexham..Jan. 25, Feb. 1-22
Alford..Feb. 1
Gainsborough..Dec. 28, Jan. 4-25
Liverpool..Dec. 14, 21, Jan. 4, 11, 25, Feb. 8-29
Bolton..Dec.21,28,Jan.11-25,Feb.1-15

CHOLERA.

Liverpool..Feb. 8.

AGUE.

Canterbury..Jan. 4, Feb. 8
Bridgewater..Dec. 14
Chatham..Dec. 7, all Jan. and Feb.
Sharnbrook..Dec. 28, Jan. 4, 25, Feb.
Wellingborough..Feb. 8-29 [1-15
Thetford..Dec. 14, Feb. 8

REMITTENT FEVER.

Teignmouth..Jan. 25
Odiham..Jan. 11, Feb. 29
Putney..Dec.21, Jan. 18, Feb. 8, 22, 29
Swansea..Jan. 18, 25, Feb. 8-29
Saffron Walden..All Dec. and Jan.
Wellingborough..Jan. 18
Thetford..Dec. 7, 21, Jan. 11, Feb. 22
Burton-on-Trent..Feb. 29
Wrexham..Feb. 15-29
Alford..Dec. 7-21, Jan. 11, 18
Liverpool..Dec.7, 21, Jan.11, 18, all Feb.
Bolton..Dec. 14-28, Jan. 18, 25

DIARRHŒA.

Teignmouth..Every week except Dec. 14 and Feb. 15
Odiham..Jan. 18, 25, Feb. 8
Bridgewater..Dec. 14 28, Feb. 1-15
Chatham..All Dec., Jan.4, 18, Feb.8-29
Putney..Dec.7-21, Jan.11-25, Feb.1-15
Wanstead..Dec. 28, Feb. 22, 29
Swansea..Dec. 21, Jan. 4, 18, Feb.
Saffron Walden..Every week [15-29
Bedford..Nearly every week
Sharnbrook..Dec. 21, Jan. 11, 25, all
Wellingborough..Jan. 11 [Feb.
Thetford..Nearly every week
East Dereham..Feb. 8-22
Pontesbury..Jan. 4, 11, Feb. 15, 22
Nottingham..Dec. 14-28, Jan. 11-25, Feb. 1, 8, 22, 29
Burton on-Trent..Feb. 8
Hawarden..Every week
Alford..Dec. 21, Feb. 22 [1, 22, 29
Gainsborough..Dec. 21, all Jan., Feb.
Liverpool..All Dec., Jan. 25, Feb. 1-22
Bolton..Jan. 18, Feb. 1, 8

DYSENTERY.

Upper Holloway..Feb. 22
Wanstead..Dec. 7
Bedford..Feb. 8
Pontesbury..Feb. 8-29
Nottingham..Dec. 14-28, Jan. 11-25,
Wrexham..Dec. 21 [Feb. 22-29
Lincoln..Jan. 25, Feb. 1
Gainsborough..Jan. 11-25
Liverpool..Feb. 8-22

TYPHUS.

Odiham..Dec. 7, 14. Jan. 11, Feb. 22
Bridgewater..Every week
Canterbury..Dec. 7-21, all Jan., Feb. 1-15 [15, 29
Chatham..Dec. 14, 28, Jan. 18, 25, Feb.
Wanstead..Jan. 11-25, all Feb.
Saffron Walden..All Dec. and Jan.
Bedford..Dec. 14, Jan. 25, all Feb.
Sharnbrook..Every week
Wellingborough..Jan. 18
Thetford..Jan. 11-25, Feb. 8
East Dereham..Dec. 21, 28, all Jan. &
Pontesbury..All Feb. [Feb.
Nottingham..Dec. 7-21, Jan. 4-18,
Alford..Feb. 22 [Feb. 11
Gainsborough..Every week
Liverpool..Every week
Bolton..Jan. 25, Feb. 1-29
Rothbury..All Jan. and Feb.

PUERPERAL FEVER.

Thetford..Dec. 21, 28, Jan. 4
Burton-on-Trent..Feb. 22
Liverpool..Dec. 21, Feb. 22, 29

CARBUNCLE.

Odiham..Dec. 28, Jan. 25
Bridgewater..Every week
Canterbury..Dec. 14-28, Jan. 11
Chatham..Dec. 28, Jan. 18, Feb. 8
Putney..Jan. 11
Wanstead..Dec. 14, Feb. 8
Swansea..Jan. 11, 18
Saffron Walden..Jan. 4, Feb, 8, 15
Sharnbrook..Dec. 7, 14, Jan. 18
Thetford..Jan. 18, Feb. 8 [Feb. 1, 8
Nottingham..Dec. 21, 28, Jan. 4, 18,
Burton-on-Trent..Feb. 22
Wrexham..All Feb.
Liverpool..Feb. 22, 29
Bolton..Dec. 14, 21, Feb. 8

---

### ADDITIONAL OBSERVATIONS.

*Teignmouth.* Mr. Lake has sent us an elaborate meteorological report of Teignmouth, for the months comprised in these returns; but we regret that want of space prevents us from publishing it in the present number. With regard to disease, he says:—

"The most prevalent form of influenza during this quarter was that in which the bronchial symptoms were slight, or almost entirely absent, together with a quick pulse, thirst, loss of appetite, alternate flushes and chills, and a general febrile state, with considerable loss of strength and depression of spirits; the most prominent feature was the severe aching pains affecting the head, trunk, and limbs. Of thirty-two cases of which I have notes, only seven presented severe or well marked bronchial symptoms; in sixteen there was catarrh, with a little cough; in twenty-two out of the thirty-two the neuralgic element predominated; in nine it existed to the exclusion of the catarrhal. These pains were also sometimes felt in the clavicular and sternal region, over the ribs, and occasionally over the sides of the abdomen, and during convalescence were often most annoying in the flexures of the knees, the calves and shins. The tongue was usually softly coated, though having a rather flabby and sodden aspect; it was seldom pointed. Nausea was frequently present, but vomiting only in three cases out of the thirty-two; diarrhœa only in one or two. The bronzing of the skin, though generally present, was not so to a great degree. Severe cases were attended by delirium; and one old woman of seventy, in whom the bronchial symptoms were severe and the nervous depression great, was much harassed in her waking state by ocular spectra. In some cases there was considerable irritation; irritation of the uterine system, especially during pregnancy, after delivery, and in other states of activity of the uterine functions. Herpes about the lips and vesications in the roof of the mouth occurred, but not so commonly as last year. The influenza was not very prevalent amongst children, nor were any of the cases that came under my notice fatal, though several were severe. The cases of scarlatina mentioned as occurring in December and January were very mild."

*Odiham.* Dr. M'Intyre says:—"The healthiness of this district has continued. No such exemption from disease during the winter months has occurred for many years. The cases that have occurred have been chiefly catarrh, influenza, bronchitis, pneumonia, neuralgia, and rheumatism; the last in several cases, in the form of rheumatic fever, severe and protracted. Three cases of acute croup occurred during the first three weeks of February. No death occurred in our parish; in the neighbourhood hooping-cough has been epidemic, but no deaths have occurred. In another parish measles had prevailed, but no case has come under my observation: several deaths have occurred from it with typhoid symptoms."

*Canterbury.* Mr. Rigden writes :—" The prevailing zymotic diseases in this city and neighbourhood during the past quarter have been principally measles and hooping-cough. About one hundred and fifty cases of the first-named disease have been under my own care, and a large number of children with this disease have not had medical attendance : it has, however, proved fatal to but nine patients during this period. Small-pox, I hope, has now left us, or more properly speaking has afflicted all those who, by neglect of vaccination, are most subject to its influence."

In addition to Mr. Rigden's report, the following communication has been forwarded to us by Mr. James Reid, of Canterbury, honorary secretary to the East Kent and Canterbury Medical Society.

*Abstract of the Report of Zymotic Diseases in the city of Canterbury during the Winter Quarter,* 1855-6.

The results are obtained from nine different observers, who furnish monthly returns of epidemic diseases for the use of the medical society ; they are embodied in the table at pp. 85-7.

" The variations of temperature during the quarter have been very considerable, from severe cold and strong easterly winds, that prevailed in the middle and latter part of December, to warm, close, spring-like weather, which prevailed for some days both in January and February.

" During the quarter the following meteorological results have been observed.

| | Dec. | Jan. | Feb. |
|---|---|---|---|
| Lowest temperature in the day time .. | 19° | 29° | 30° |
| Highest .......................... | 47° | 49° | 50° |
| Mean.............................. | 34° | 39° | 40° |
| Lowest temperature during the night.. | 18° | 26° | 28° |
| Highest .......................... | 45° | 49° | 47° |
| Mean.............................. | 31° | 36° | 37° |
| Lowest point of barometer .......... | 29·20 | 28·87 | 29·61 |
| Highest .......................... | 30·17 | 30·32 | 30·35 |
| Mean.............................. | 29·73 | 29·41 | 29·88 |
| Amount of rain ..................... | 2·32 | 2·07 | ·91 |

" The total amount of rain during the year ending December 31st, 1855, at Canterbury, was 23·37 inches.

" During December the prevailing winds were W. to N. During January the prevailing winds in the first half of the month were E. to S. and N. equally, during the latter half W. to S. During February the prevailing winds were W. to S., and next in order E. and N.E. There was considerable fog and mist on several days this month.

" The most prevalent zymotic disease has been measles ; two hundred and thirteen cases have been returned. In the summer quarter of 1855 measles occasionally occurred, the disease having appeared in June amongst some troops recently marched from Chatham. In the early part of the autumn, also, cases were observed; but suddenly, on the 25th of November, the disease became epidemic and spread to all parts of the town. The return for the month of February is the highest for the three months. The mortality in the cases returned is 3·75 per cent. The complications of the disease have been pneumonia and pertussis, but in proportions to give the epidemic at present a mild character. Many cases are not seen by medical men. The disease likewise prevails in the surrounding villages.

"The next disease in order of frequency has been small-pox, which has been epidemic about twelve months, and now appears to be gradually subsiding. Thirty cases have been reported during the quarter, the rate of mortality in these being 13·33 per cent. The fatal cases all occurred in unvaccinated subjects; of those attacked with the disease 36·6 per cent. were not vaccinated, and 63·3 per cent. had been vaccinated. Several of the vaccinated had the disease in a confluent form, but, for the most part, they had it in a much modified degree. Two instances occurred of the co-existence of small-pox and the vaccine disease. In one, variola appeared on the ninth day after vaccination, in a confluent form, but was influenced by the vaccine disease: it was reported to have been three days slower in its progress than the natural disease. In the other instance small-pox appeared on the tenth day after vaccination; the vaccine disease having matured thoroughly, the varioloid disease was quickly modified, the pustules in various degrees of development shrivelled on the sixth day, and speedily vanished. An instance is mentioned of varioloid fever followed by no eruption, though pain in the head persisted long after the usual period of the primary fever. The subject of this attack had small-pox in childhood, and small-pox prevailed in the family at the time of the present illness. One of the fatal cases occurred in a child six days after birth, the mother having had the eruption of modified small-pox two days after giving birth to the child.

"Scarlatina, which has prevailed since the autumn of 1853, appears to have gradually subsided—indeed, it may almost be considered to have returned to a sporadic condition: six cases are noticed, one of which was fatal from bronchitis.

"The mortality in fever has been at the rate of 6·25 per cent.

"Pertussis has been more prevalent, and has been complicated with broncho-pneumonia and rubeola: the mortality has been 5·88 per cent.

"Erysipelas has been rather more prevalent, principally idiopathic of the head and face: nearly half the cases returned occurred in the hospital."

*Chatham.*—Dr. F. J. Brown writes: "The direction of the wind was observed on eighty-two days out of the ninety-one, in the quarter ending 29th of February. It was east (either due or conjoined with another wind), on twenty-seven days. There was haze or fog on ten days out of the ninety-one. The weather was exceedingly cold in the early and middle portions of December: since then, it has been unusually warm.

"Catarrh and influenza have been prevalent; also diarrhœa and biliary disorder. There has been a great amount of erysipelas. Scarlatina entered the town in January, and has hitherto confined itself to one portion, for the most part. Paludal fever, in the shape of eyebrow ague, has been very common. Hæmorrhagic cases have been unusually numerous; the list including apoplexy, bleeding at the nose, spitting and vomiting of blood, bright intestinal hæmorrhage, and uterine hæmorrhage. One case of typhoid fever, in a boy aged 11 years, deserves mention. On the fourth day of the disease, hæmorrhage from the intestines occurred suddenly, to the amount of eight ounces; and again on the sixth day, and half that quantity on the seventh day. The fever turned on the fourteenth day. I treated the case with acetate of lead and acetic acid, cold beef tea, sulphuric ether, and Dover's powder; and I applied a blister over the right iliac region. Two cases of painful erythema have occurred in the form of red patches of great extent over the body, succeeded by thin and extensive cuticular exfoliation."

*Putney.*—Mr. Whiteman says: "The temperature of the last three months has been so variable as to be productive of a large amount of disease in this district. Ozone has varied very greatly in amount. Catarrh and influenza have been very prevalent. A few cases of scarlatina were observed at the latter part of December; and what is very unusual in January and February, diarrhœa of a rather severe character attacked persons of all ages, amongst the poor especially. Hooping-cough, however, has been the prevailing epidemic for the last two months, scarcely a family where there were young children escaping. Happily, it has not been very fatal. Out of some fifty cases of considerable severity, two patients only have died, and those were scrofulous children. Two cases of small-pox in adults were treated about the middle of February, both the patients having been successfully vaccinated when young.

"A comparison of the rate of mortality amongst the poor at the two periods of 1844 and 1854, shows a greatly improved state of the public health in this district, which is highly satisfactory.

"Through the assistance of the registrar of the district, I was enabled to make an analysis of the deaths in this parish in the year 1844. I fixed upon that year, chiefly because it seemed to be a period in which no particular epidemic had prevailed. I find in that year, that eighty-nine deaths took place in Putney. Of this number, fifty-one were males and thirty-eight were females. Of the total number, considerably more than one-third were children under four years of age. These eighty-nine deaths were distributed as follows:—

| | | |
|---|---|---|
| Amongst families of gentry and professional men . . . . . | 11 | 89 |
| Amongst tradespeople - . . | 12 | |
| Amongst the labouring classes . . | 66 | |

"It will be perceived, then, even though we allow the labouring classes to have out-numbered the gentry and tradespeople in the proportion of two to one, the mortality will have been most excessive amongst the poor in this year.

"At the close of the year 1854, a similar calculation to the above was made. The sickness amongst the poor in this parish has increased considerably with the increase of population; but the mortality amongst the same class has diminished in an inverse ratio with the increase of population; and this, it is believed, is owing to the adoption of many wise sanitary measures and improvements since 1844. The sixty-six deaths amongst the labouring classes out of the eighty-nine which occurred in 1844, must be looked upon by all as being excessive on the side of the poor. Ten years afterwards, viz., in 1854, only *thirty-five* pauper deaths took place out of 1363 cases of pauper sickness! This gives 2·5 per cent. of deaths only in that year, although it was one in which a serious outbreak of cholera occurred."

*Wanstead.*—Dr. Collins writes: "A typhoid fever, running into typhus, has prevailed during the past two months; it has been confined chiefly to one spot, which is crowded with badly-drained, ill-ventilated huts, situated in the Grove: three deaths out of about fifty cases coming under my treatment have already occurred; and there have been many fatal cases within three miles."

*Swansea.*—Mr. Michael says: "Isolated cases of scarlatina continue, also of measles; but they are so slight as scarcely to demand treatment. There is an almost entire absence of disease; and cases of illness generally recover. There has been very fine weather during the greater part of the winter quarter."

*Saffron Walden*—Mr. Spurgin writes that "Rheumatism and bronchitis have been the prevailing diseases of the quarter. Cases of typhus and remittent fevers have been frequent in the neighbourhood. On the whole, however, the season must be considered healthy. Scarlatina and hooping-cough are on the decline, the former having now nearly disappeared. Diffused cellular abscesses and ecthyma have been unusually prevalent, but are now declining. Influenza has been common among horses. Prevailing wind, westerly. Vegetation backward."

Mr. Stear also says: "There has been, during the last three months, a remarkably small amount of illness—about one-third as compared with the correspondent months of last year. The poor in my district have been very healthy: for more than a month, not a single death occurred in a population of 6000. Hooping-cough is very prevalent. The weather is very changeable, but generally mild for the time of year; and the wheat is looking well."

*Bedford.*—Dr. Barker says: "During the past quarter, cases of diarrhœa and influenza have been numerous: small-pox, principally a modified form, has appeared in some families in Bedford. Severe cases of fever have occurred in the neighbourhood, during February and the latter part of January. The entire quarter has been characterised by remarkably changeable weather."

*Sharnbrook.*—Mr. Stedman says: "Typhus has been unusually prevalent, but has been principally confined to the village of Felmersham (in a straight line, about three-quarters of a mile from Sharnbrook), situated on the right bank of the river Ouse. During the last eight months, nearly 100 cases of typhus have occurred in that place and its neighbourhood, under my own observation: many of the cases have been complicated with diarrhœa. In two cases only has death occurred. In another village, out of my own district, higher up the river, but on the same bank, typhus has also been prevalent."

*Thetford.* Mr. Bailey says: "Scarlatina continued very prevalent throughout the town and neighbourhood, to the middle of January, when, during four weeks, no new case occurred; but from the middle of February to the end, several cases came under treatment. It still bore its mild type, requiring but little medical aid. A fatal case occurred in a very young infant. Measles was very prevalent throughout the quarter, attacking all ages. In some cases the inflammatory symptoms were very severe, and the cough most intense, requiring active depleting treatment. This state, I have no doubt, was produced by the atmospheric changes. The month of December proved considerably below the average; and the difference of temperature only in a few hours was 26° and 30°. Such a sudden transition had a serious effect upon the human system, especially on those affected with pulmonary diseases. Not only measles, but hooping-cough (which continued epidemic), became very severe in their symptoms, requiring blisters, etc., prolonging the disease, and occasioning excessive debility. Catarrhs and influenza have been more general than in last quarter; the symptoms more severe, and the cough and sense of suffocation, in many cases, very distressing. In the aged, and in young children, fatal cases have taken place. The vicissitudes of heat and cold which have been experienced during this quarter have given rise to numerous cases of diarrhœa, both in old and young subjects, producing extreme prostration of power; in some instances, nearly approaching the choleraic character. Children were more affected with it than adults. Many cases of cynanche tonsillaris have occurred in the last two months, arising from imprudent exposure, and accompanied by smart febrile symptoms: some cases terminated in abscesses of the tonsils. Typhus fever came under our notice in four cases, occurring in labouring men, and running a long course. Two cases were followed by abscesses, and dropsical effusions of the lower limbs. One case of puerperal fever occurred, which terminated fatally after five days: the labour was natural, and only of four hours duration; no milk was ever secreted, and the lochial discharge was not suppressed until the day before death. No other case has occurred, although I have attended many cases of labour since. This is the fourth case I have attended during forty-five years practice, and the second death. Owing to the mild weather in the last month, the diseases have been but few: only three cases of scarlatina and two of measles. Hooping cough has entirely left the town. A few cases of rheumatism, both chronic and acute, have come under notice. Catarrhs and influenza have been less frequent. Such a paucity of disease can only be accounted for by the equal pressure of the atmosphere, the absence of wind, the low state of the hygrometer during the month, indicating the dry state of atmosphere and the little rain fallen, not amounting to three-quarters of an inch. The diseases which are uninfluenced by weather, and under medical treatment, were—paralysis, nephritis, cystorrhœa, iritis, dyspepsia, gout, hydrocele, syphilis, leucorrhœa, menorrhagia and cephalalgia, with some cutaneous diseases. I find, upon inquiry of our veterinary men in the neighbourhood, that influenza has been very prevalent with horses and cattle—enteritis and pneumonia also. There has been less disease among sheep.

"The following record of deaths has been extracted from the Registrar's Book :—

| *December.* | | *January.* | | *February.* | |
|---|---|---|---|---|---|
| Decay of nature.... | 1 | Measles .......... | 5 | Decay of nature .... | 1 |
| Debility .......... | 4 | Decay of nature.... | 1 | Debility .......... | 1 |
| Paralysis ........ | 1 | Puerperal fever .... | 1 | Measles .......... | 1 |
| Nervous exhaustion | 1 | Pneumonia ........ | 2 | Atrophy .......... | 1 |
| Measles .......... | 4 | Typhus .......... | 1 | Consumption ...... | 3 |
| Convulsions ...... | 1 | Diarrhœa ........ | 2 | Dropsy............ | 1 |
| | | Hepatitis.......... | 3 | Varicella .......... | 1 |
| | | Consumption ...... | 5 | Hydrocephalus .... | 1 |
| | | Hooping cough .... | 1 | Bronchitis ........ | 1 |
| | | Debility .......... | 1 | | |
| | | Convulsions ...... | 1 | | |
| | | Endocarditis ...... | 1 | | |
| | 12 | | 24 | | 11 |

*East Dereham.*—Dr. Vincent says: "Most of the cases of scarlatina have been mild. Typhus has been prevalent in the villages around Dereham, and I have been able to trace its progress from the house where it originated, to the houses of brothers and other relations, who have had communication with the party first attacked. In many instances, the cottages where it occurred have been the cleanest and best placed in the village."

*Burton-on-Trent.*—Dr. Spencer Thomson writes: "One fatal case of scarlet fever occurred in a woman, aged forty-five, who was seized two or three days after her confinement. One of her children had just passed through the disease, in a form apparently mild. Throughout February, catarrh, influenza, and bronchitic attacks have been unusually prevalent, especially a few days after the weather became mild."

*Wrexham.*—Dr. E. Williams states that acute rheumatism was prevalent during December and the early part of January.

*Alford.*—Dr. West says: "Acute rheumatism has been very prevalent during the past quarter, as also intermittent neuralgic affections. The season has been very rainy."

*Gainsborough.*—Dr. Mackinder writes: "The diseases of this quarter have been of an adynamic type, and required a more than usual amount of stimulants. Of croup, erysipelas, and diarrhœa, there have been a few cases; many of catarrh, bronchitis, and typhoid; and hooping-cough has been epidemic. In a neighbouring village, there has been a case of small-pox after vaccination in an adult. I have had several cases of chronic inflammation of the inguinal glands, some terminating in resolution, others in suppuration. The patients were of different sexes, and their ages varied from 17 to 60. Except in one or two cases, no tangible cause could be assigned."

The subjoined meteorological report has been furnished by Mr. Dyson. "The weather, during the quarter ending Feb. 29th, has been peculiar. The month of December was unusually cold, the average of the month being little above the freezing point, and on the night of the 22nd the temperature fell to 21° of Fahrenheit. Fog was prevalent, and snow fell on the 6th, 7th, 8th, 9th, and 10th. The Trent was frozen over for several days, commencing on the 21st. Very little rain fell, the rain and snow together producing water only to the depth of half an inch. With the exception of a few days at the beginning, January was a cold month, and the Trent was a second time closed by ice; very little snow fell; rain nearly 2½ inches. February was particularly mild, but the sky for the greater part of the month covered with cloud. The amount of rain which fell was less than an inch. The amount of ozone developed during the quarter was insignificant. In December, the greatest heat was 48° on the 14th; the greatest cold, 21° on the 22nd. In January, the maximum of heat was 50° on the 24th, and the minimum 22° on the 13th. In February, the highest range of temperature was 55° on the 9th, the lowest 26° on the 2nd. Several meteors were observed; one was especially brilliant on the 3rd. Lunar halos, displaying the prismatic colours, were prevalent from the 13th to the 17th of February. The quarter is remarkable for the intense cold during the first half, and for the mildness of the second."

*Liverpool.*—Mr. Bickerton makes the following report. "Dec. 7th, 1855. Scarlatina, measles, and hooping-cough, prevailed in an unusual degree. During the week there were 74 deaths from these three diseases alone, viz., scarlatina, 38; measles, 20; hooping-cough, 16: also from typhus, 8; small-pox, 1; syphilis, 2. The deaths from measles were more frequent than in any week of the previous 17 months. Scarlatina had been epidemic nearly four months, and had destroyed upwards of 500 lives. Diseases of the lungs caused 82 deaths. The total number of deaths in the week was 257; 173 were children, of whom 154 were below the age of five years. I attended one case of confluent small-pox in an adult, 22 years of age, who never was vaccinated; he died from pneumonia, on the twenty-first day.

" Dec. 15th, 1855. 274 deaths were registered from all causes during the week, being 31 more than the corrected average for the same week in previous years. Scarlatina caused 21 deaths; hooping-cough, 23; measles, 15; typhus, 7; small-pox, 3 (1 after vaccination); syphilis, 1. The mean temperature for week was 36, being 7½ below the average of the same week last year.

" Dec. 22nd, 1855. 244 deaths from all causes occurred during the week, being 11 above the corrected average for previous seven years. Zymotic diseases caused 67 deaths; viz., scarlatina, 17; hooping-cough, 15; typhus, 5; small-pox, 1; measles, 15. Inflammatory diseases of chest caused 59 deaths, and phthisis 33. The effect of the severe weather was chiefly felt by infants and children; 141 deaths occurred in children below 5 years, and 33 in persons above 60.

" Dec. 29th, 1855. The number of deaths was 244, being the same as in the previous week, and about equal to the corrected average of the corresponding week for previous seven years. The deaths in the parish were 164, and 80 in the out-townships. The deaths from zymotic diseases amounted to 68, viz., from scarlatina, 24; hooping-cough, 14; measles, 11; typhus, 4; small-pox, 1. Inflammatory affections of the lungs caused 65 deaths; phthisis, 21.

" During the quarter ending 29th Dec. 1855, 3,231 deaths were registered in the borough, including 157 on which inquests were held; being 100 less than the corrected average of the same quarter of the preceding eight years. The deaths from zymotic diseases were 200 more than the average, owing to the prevalence throughout the entire quarter of scarlatina, which occasioned 447 deaths. Measles caused 155 deaths; hooping-cough, 152; diarrhœa, 112; typhus, 96; tracheitis, 43; syphilis, 24; small-pox, 15. No death took place from cholera, this being the second quarter in which such absence has occurred during the last eight years.

"Jan. 5th, 1856. 218 deaths occurred in the week from all causes, the corrected average for the previous eight years being 250. Diseases of lungs for previous four weeks had been respectively 107, 92, 86, 74. Corresponding with this decreasing mortality, there had been an increase in the temperature. The mean, which the two previous weeks averaged more than 8 degrees below that of the same period of former years, was last week more than 2 degrees above it. Hooping-cough caused 24 deaths; scarlatina, 21; typhus, 6; measles, 5; small-pox, 2; syphilis, 2.

"January 12, 1856. 228 deaths occurred from all causes; being 22 less than the corrected average for previous eight years. The deaths from zymotic diseases were, from scarlatina, 28; hooping-cough, 20; measles, 9; typhus, 3; small-pox, 2. 66 deaths were caused by diseases of lungs. Of the 228 deaths, 128 were below 5 years, and 22 above 60.

"January 19, 1856. In consequence of the cold which had prevailed of late, there was a general increase of mortality for this week. The deaths from all causes were 252, the corrected average for former years being 204. The whole excess is accounted for by deaths occurring in chest affections, which were 98; being 66 in the previous week. There were from scarlatina, 24 deaths; hooping-cough, 22; measles, 7; typhus, 7; small-pox, 1 (previously vaccinated).

"January 26, 1856. 227 deaths were registered during the week; being 12 less than the corrected average of previous 7 years. Zymotic diseases. Sscarlatina, 13; hooping-cough, 20; measles, 7; typhus, 4; small-pox, 2. Diseases of lungs and consumption caused 99 deaths. A gentleman died from delirium tremens. 129 died below the age of 20; 73 between 20 and 60; 25 between 60 and 90.

"February 2, 1856. The mortality for this week was lower than in any week of the preceding three months. The deaths from zymotic diseases were fewer than for six months, and from typhus fewer than for twelve months previously. The deaths from all causes were 214; being 144 in the parish, and 70 in the out-townships. The corrected average of the same week of the seven years preceding was 286. Hooping-cough caused 23 deaths, scarlatina 12, measles 3, typhus 2, diarrhœa 5, cholera 1. From diseases of the lungs the deaths were 75; being 24 less than in the previous week. Rheumatism caused 3 deaths.

"February 9, 1856. The cold weather during the week caused an increase in the mortality. The deaths from all causes were 240; being 6 less than the corrected average for the previous seven years. Diseases of lungs caused 98 deaths, scarlatina 16, hooping-cough 17, diarrhœa 6, measles 2, typhus 2, small-pox 2 (unvaccinated). The mortality from fevers generally was unusually low. Delirium tremens caused one death; and a married woman, aged 37, died from the continued use of opium. The temperature was 6 degrees below average, but increased latterly 13 above it.

"February 16, 1856. In consequence of the mild weather which prevailed from the 6th to the 16th of February, there was a decrease in the mortality. The total number of deaths registered in the week was 205; being 34 less than the corrected average of previous eight years. From zymotic class of disease there were—scarlatina, 17; hooping-cough, 10; diarrhœa, 8; measles, 4; typhus, 4; small-pox, 2; syphilis, 1; mumps, 1; and 85 from diseases of the lungs.

"February 23, 1856. 243 deaths were registered; being about the average, but above last week's report. Hooping-cough caused 14 deaths, scarlatina 12, tracheitis 6, measles 3, syphilis 2, varicella 1, typhus 5, small-pox 2, influenza 1, and erysipelas 1. Diseases of lungs caused 82 deaths. The unusual number of 27 infants died from convulsions and teething; and delirium tremens was fatal to 2 males."

*Bolton.*—Mr. Pendlebury writes: "Scarlatina has evidently been rather rapidly declining since the middle of January. The cases at present on hand are chiefly of the milder type, but very frequently are complicated with some pulmonary affection. Idiopathic erysipelas of the head and face was particularly prevalent during January and a portion of February, whilst the weather was rainy and the atmosphere cold. Although the majority of cases were very severe, yet they yielded readily to iron, or iron combined with quinine; thus proving their asthenic nature. There have been a few cases of continued fever, with gastro-enteric symptoms. The mortality has been somewhat less than in previous years,

*Rothbury.*—Mr. Summers says: "The weather during the past quarter has been changeable, but, on the whole, rather mild for the season; and at present there is every appearance of an early spring. As far as my knowledge goes, there has been but a single case of measles (fully developed), and the same of croup; but throughout the quarter catarrh has been epidemic, amongst both adults and children; and in numerous cases amongst the latter, it has seemed to assume croupy character, whilst many others have presented all the symptoms of measles except the eruption. Typhus is still prevailing, of a mild type generally,"

